# The deployment of a tissue request tracking system for the CHTN: a case study in managing change in informatics for biobanking operations

**DOI:** 10.1186/1472-6947-10-32

**Published:** 2010-06-02

**Authors:** Mary E Edgerton, William E Grizzle, M Kay Washington

**Affiliations:** 1 Section of Pathology Informatics, Department of Pathology, MD Anderson Cancer Center, Houston, TX, USA; 2 Department of Pathology, University of Alabama, Birmingham, AL, USA; 3 Department of Pathology, Vanderbilt University Medical Center, Nashville, TN, USA

## Abstract

**Background:**

Managing change has not only been recognized as an important topic in medical informatics, but it has become increasingly important in translational informatics. The move to share data, together with the increasing complexity and volume of the data, has precipitated a transition from locally stored worksheet and flat files to relational data bases with object oriented interfaces for data storage and retrieval. While the transition from simple to complex data structures, mirroring the transition from simple to complex experimental technologies, seems natural, the human factor often fails to be adequately addressed leading to failures in managing change.

**Methods:**

We describe here a case study in change management applied to an application in translational informatics that touches upon changes in hardware, software, data models, procedures, and terminology standards. We use the classic paper by Riley and Lorenzi to dissect the problems that arose, the solutions that were implemented, and the lessons learned.

**Results:**

The entire project from requirements gathering through completion of migration of the system took three years. Double data entry into the old and new systems persisted for six months. Contributing factors hindering progress and solutions to facilitate managing the change were identified in seven of the areas identified by Riley and Lorenzi: communications, cultural changes in work practice, scope creep, leadership and organizational issues, and training.

**Conclusions:**

Detailed documentation of the agreed upon requirements for the new system along with ongoing review of the sources of resistance to change as defined by Riley and Lorenzi were the most important steps taken that contributed to the success of the project. Cultural changes in tissue collection mandated by standards requirements introduced by the Cancer Bioinformatics Grid (CaBIG^®^) and excessive reliance on the outgoing system during a lengthy period of dual data entry were the primary sources of resistance to change.

## Introduction

In 2006, the Cooperative Human Tissue Network (CHTN) migrated from a distributed model of six stand-alone FoxPro databases to house their tissue requests, in use for almost 20 years, to a centralized web-based system with an Oracle backend relational database. As a result, their information collection process, the ontology and terminology for disease specification, histopathological characterization, and tissue preparation procedures and reagents were not only standardized across the divisions, but they were made compliant with ongoing standards initiative within the National Cancer Institute (NCI).

Migration to this new system had a major impact on their data management and workflow. A significant source of change was the new ontology and terminology for the disease and histopathological qualities of the tissues being requested that was introduced in order to achieve compliance with the NCI standards initiatives. As a case study in change management, this migration touched on almost every aspect of change, from hardware to software engines through data models, ontologisms, and terminology. Similar to previous work in presenting cases of change management (e.g. [1]), we use the classic paper by Riley and Lorenzi [2] to dissect the problems that arose, the solutions that were implemented, and the lessons learned. With tissue resources receiving recognition as a fulcrum of translational research, the lessons learned here have relevance for the migration of scores of tissue banks as they consider using tools such as caTissue, distributed by the Cancer Bioinformatics Grid (caBIG^®^) under the auspices of the NCI [3]

## Background

The CHTN, founded by the NCI in 1987 to distribute human tissues to researchers across the United States, provides a uniquely configured prospective tissue collection service [4,5]. Investigators apply to one of a set of six networked divisions that service geographically defined regions across the US. Individual investigators are allowed to specify the anatomic site, histopathology or concurrent disease, tissue preparation method, and reagents for use in tissue preparation in order to match pre-analytical variables to their research requirements. If any one division cannot satisfy the tissue request of an approved investigator following a reasonable amount of time (approximately two weeks) then that tissue request is networked to the other divisions.

The advantages of the prospective mode are two-fold: 1) tissue collection procedures and reagents are optimized to the needs of the experiment, and 2) information that is shared across the network is limited to the tissue request, thus elegantly sidestepping issues associated with sharing tissue repository information that may contain protected donor information.

Until 2007, the CHTN relied upon distributed stand-alone instances of a FoxPro database with a visual basic interface to store investigator's tissue requests. In order to network tissue requests that could not be served locally, each division would mark specific tissue requests for networking within their own databases. Every night, a coordinator (hereafter referred to as coordinator) who functioned in the role of resource manager at each division would upload their individual database to a central FTP server. The networked tissue requests with project and investigator details were merged across the divisions, joined to each submitted database, and returned to each site in the morning.

Several problems arose over time. The information in the individual databases exceeded the capacity of FoxPro, and corruptions were introduced at the local level. These corruptions were disseminated to the other divisions during the networking process. Tissue requests became more complex, with investigators specifying multiple tissue preparation methods for a single tissue type. These additional details were relegated to unstructured comment boxes and entered in free-text format. There was no consistent choice of the location where this information would be placed, and there were no standards in the actual values used for the entries. Basically, the method for storing the information was dependent upon the data entry personnel at the individual divisional level. Thus, important data was distributed into various unstructured storage locations in the database using terminology and styles that varied within and across the divisions. To address these problems, the CHTN invested in the development of an updated FoxPro database to be implemented as a distributed system. Between approximately 2000 and 2004 they contracted with an outside developer to develop an updated FoxPro based system that would include more data elements. However, requirements were not well-defined and the software never reached production use by the CHTN secondary to scope creep, changes in software development personnel, and organizational issues.

In 2004, the NCI began the caBIG^® ^initiative to facilitate data sharing. The data models and terminology of the CHTN were based on a set of terms constructed by the pathologists for use by the coordinators and other non-medical personnel more than a decade earlier. Thus, the CHTN, an important tissue collection resource for the NCI, was faced with the need to update its ontologisms and terminologies in order to be compliant with NCI standards.

**Figure 1:**
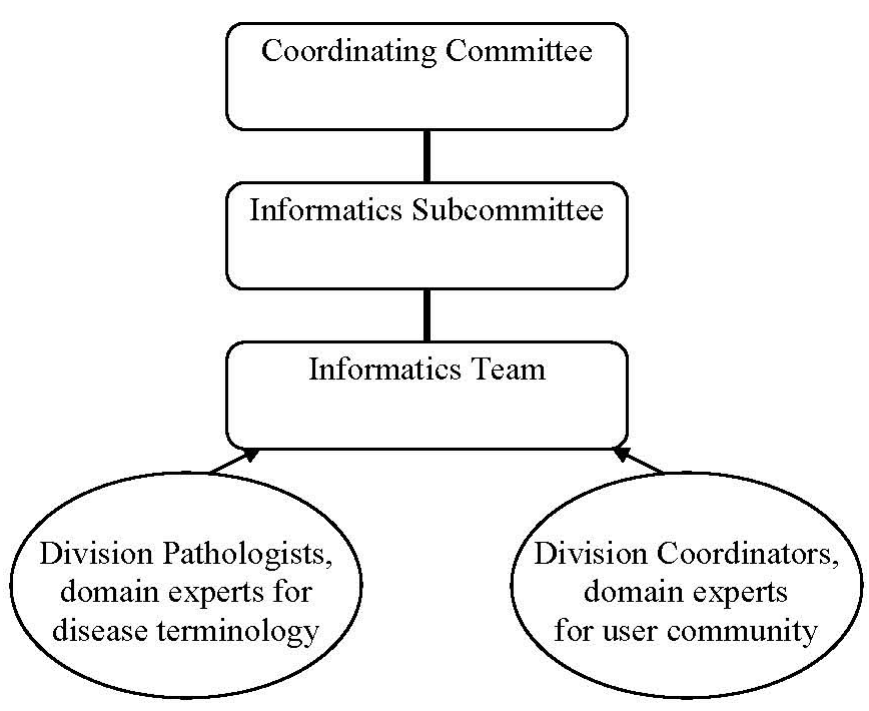
**Leadership structure**.

In that same year the CHTN decided to consider exploring the option of a centralized database with browser based access instead of continuing to pursue the FoxPro project. The organizational structure required that this be put to a vote before the steering committee that provides governance to the six divisions that comprise the CHTN, which is known as the Coordinating Committee. In spring, 2004, the Coordinating Committee of the CHTN approved a motion to develop a new web based investigator/tissue request management system to solve these problems. An Informatics Team from the Western Division, housed at Vanderbilt University, was charged with constructing a new system that would retain the functionality of the old system and address the problems just described. The Informatics Team consisted of a Pathology Informatician as Director and an Information Science Analyst acting as both business analyst and programmer. Coordinators across the divisions were consulted as domain experts for required functionality and use of data elements. Pathologists in the divisions were consulted as domain experts for the anatomic site and disease specific terminology to be used. The Informatics Team would report to the Informatics Subcommittee, which in turn reported to the Coordinating Committee. The Informatics Subcommittee was charged with evaluating the progress of the project and making final recommendations to the Coordinating Committee on migration to the new system. The Informatics Team was charged with gathering the requirements from the coordinators across the network, evaluating the data elements and design features of the FoxPro upgrade project to determine those that would be retained, formulating compliance with caBIG^®^, and finally developing the system. The Informatics Team would also be responsible for migration of the legacy data in the old FoxPro databases from all of the network divisions into the new database. Figure 1 depicts the leadership structure for the project and the relationship to domain experts.

This migration to a centralized system involved changes that spanned culture, ownership, and technology. There was 1) a change in local ownership of data to a centralized database, 2) a change in the computer-user interactions from a locally installed database and front-end to a web-based interface, and 3) a change in the terminology and ontologies to be used in order to become compliant with NCI terminology. The classic paper by Lorenzi and Riley on managing change was used to guide the planning, evaluation, and implementation process in order to successfully manage the ongoing changes. Lorenzi and Riley list nine common reasons for contemporary systems failures. These are ineffective communication between developers and users; cultural issues, including hostility from the user community toward the developer and a lack of a strategy to grow a new culture; underestimation of the complexity of the problem to be addressed; scope creep based on a failure to clearly define objectives and requirements or to negotiate new requirements that arise; organizational issues based on an ineffective reporting structure and a lack of a vision for change; technology issues arising from systems that attempt to capture the leading edge of technology; lack of sufficient training; and leadership issues leading to user groups sensing a lack of ownership of the process.

## Design and Development Process

### Requirements Specification

The project was initiated with a two-day workshop in June, 2004 to define the minimum set of data elements and the minimum functionality required before the CHTN would migrate to a new product. The divisions each sent at least one person at the coordinator level to the workshop.

The Informatics Team used an approach equivalent to a Level 3 Capability Maturity Model (CMM) for software engineering where data elements, relationships, and business transactions that were shared across all of the divisions were defined [6]. The Informatics Team used the data dictionary and functionality defined for the FoxPro update project as a starting point. Data elements and minimum functionality were reviewed and approved. Two rules were applied to mandate standard procedures across the divisions. If changes in a particular procedure would not have a major negative impact on any single division's ability to meet an investigator's tissue request, then majority vote prevailed. If a single division justified a claim that a particular change was critical to their internal procedures, then 100% of the vote was required to approve the data element or function. As a result, changes in data elements typically required a majority vote while changes in functionality could require a unanimous vote. This approach to delineate critical requirements from local preferences was used repeatedly during the overall process of change management.

### Software Development

The Agile programming methodology was employed. Initial focus was on the data model and the design of the web-based input forms and less on the terminologies employed. Delays in progress were encountered at the start due to personnel changes in the business analyst/programmer position in early 2005. In fall 2005, a prototype was presented at the semi-annual coordinating committee meeting. In the course of this presentation, the Western team realized that the network did not yet have a standard model for creating tissue requests. For example, each division had the freedom to enter a tissue request for tissue from multiple sites from a single donor as one tissue request for multiple sites, with the additional sites listed in a comment, or as multiple tissue requests, one for each site. Similarly, tissue requests for diseased and normal tissue, where a single donor was not required, could be entered as one or multiple tissue requests. These discrepancies across the network were not anticipated in the June 2004 planning meeting. Using the CMM Level 3 equivalent approach, the community came together and defined a single data model for organizing tissue requests.

At this meeting, acceptance of the interface was noted to have a cultural bias. Users who were accustomed to HTML based forms presented by web browsers, with transitions to new forms and pop-ups, found it easier to navigate the system. Users who were not acculturated to HTML based forms were reticent to leave the format of the stand-alone system. A majority vote was taken and it was decided that the browser based system would be used.

A new version, with the data model in use today, was introduced for evaluation at a special one-day Informatics Subcommittee face to face meeting in January 2006. Functionality was reviewed against the requirements. Scope creep, an ongoing problem, was directly addressed as the community strived to separate critical from desirable changes to the system to finalize a product that could be implemented. Variation in workflows and cultural acceptance of browser based forms were the major difficulties encountered in finalizing a list of changes.

A key problem identified in this meeting was the difference in reports used by each division. Tissue request reports are driven by local workflow. The coordinators, who represented the primary user group with the greatest risk exposure for the migration, were given the task of deciding upon a common set of data elements and a common format for these reports. The remainder of the programming tasks were defined and given as a work list to the Informatics Team.

The group met again in May 2006. All of the items from the work list were addressed. However, the divisions had not been able to agree upon a common report to drive tissue collections. It became clear that a standard report was not forthcoming. The Informatics Team devised a solution consisting of two reports: one where the data element set was the union of all data elements suggested, and a second where the data element set was the intersection of all of the suggested data elements.

It was agreed that the final terminology should be developed. The processes of migrating from the old system to the new system were defined with a target date of September 2006. The divisions as a whole decided that they would prefer to perform dual data entry into both systems for one month following the data migration in case the new system was not adequately managing the data. An additional programmer was hired by the Informatics Team at the Western Division to develop migration software.

The task to develop the final terminology for the anatomic sites disease lists with histopathological classification was assigned to Mary E. Edgerton as the senior pathology informatician on the team. She was charged with organizing a list of data elements and their relationships (syntactics) and the allowable values for each data element (semantics). This list was circulated to a pathology list-serve with membership consisting of a pathology representative from each division. Cancer diagnoses terms were based on the College of American Pathologists Cancer Protocols [7] also used by caBIG^®^. Non-neoplastic ontological structure was developed de novo by Edgerton with approval by the remainder of the pathologists across the network [8]. Options to accept any kind of tissue or any abnormal type of tissue were retained based on the original lists developed by the CHTN.

## Migration Process

Once these anatomic sites and diseases lists were approved by all divisions, Dr. Edgerton created a map from each of the previously used terms from the old to the new system. This map was distributed and approved by the pathologists from all of the networks. A small number (less than five) of diagnoses in the old system were not recognizable and were not considered amenable to mapping. The coordinators at each division were notified of these and asked to work with their local pathologists to replace these with terms that were amenable to mapping. A controlled vocabulary was defined by the coordinators across the divisions for the remaining data elements that describe the investigators, investigator institution type, funding sources, bill payment methods, shipping resources, and preparation details.

The process of mapping these terms took longer than anticipated and the migration date was pushed back to late October. The process was slowed when the various divisions realized that their data would have to be made available to the Informatics Team, who were situated at the Western Division, for clean-up and preparation for migrating into the new system. The other divisions had not realized that they would be sending their tables to a programmer who worked for the Western Division for this task and were reticent to do so. This reluctance in sending the data for the purposes of migration demonstrated the ownership issues that made the transition to a central system difficult for the community to accept.

In October 2006 the CHTN met for its fall semi-annual meeting and reviewed the system. It was agreed that each division would send its data to the Informatics Team at Western as of 5 pm CST on Friday December 1. The Western Division was given one week to complete migration of this data to the new system. During this time, each division agreed to continue to enter new data into the legacy system, and keep a paper copy for entry into the new system once data migration was completed.

The network agreed that when the data migration was complete, they would use the new Tissue Request system for all new investigators and tissue requests, and for modifications of existing tissue requests. It was also agreed that for one month, the network would perform dual data entry using the old and the new system in order to insure that the new system fully met their needs and was sufficiently error-free.

The Informatics Team at Western advised each Division that they would have to curate the migrated tissue requests. This was necessary because critical information had been entered as unstructured text in comments. For example, adult polycystic kidney disease (AKPD) was not in the existing CHTN disease list. A request for APKD had been entered as a normal kidney request with a comment that lesional tissue from a patient with APKD was requested.

In order to assist the coordinators, who would be performing this curation, a tissue request attribute with values of Mapped, Curation in Progress, and Curated was added. Coordinators could create work lists based on the curation status of the tissue requests to guide their curation process.

## Post-Migration Experience

Data was migrated by the end of the first week of December 2006 with a timetable of one month to end dual entry, and completely transition to the new system. However, it became clear that in the early post-migration experience, it was much easier for users to rely on the old system that to learn the new system. It was also clear that the divisions had not realized the extent of curation that was necessary to correct the legacy data in the new system. Therefore they needed to be able to use the old system until the data in the new system was corrected. This problem worked to prevent a rapid transition and several divisions fell behind in dual entry.

The Informatics Subcommittee recognized that the problem of curating the tissue requests was having a negative affect on efficient transitioning. A series of teleconferences for the coordinators were held to discuss a timetable for finalizing the curation process. Even then there was a reluctance to end dual entry. Finally, in late February, a problem that had not been anticipated was identified. Write privileges for payment and billing information, attributes that are associated with an investigator, were restricted to the primary division to which an investigator applied, even for networked investigators. In practice however, the divisions might set up their own individual payment arrangement with a networked investigator. This issue had not been identified in June 2004 as a requirement. In the coordinators' experience this requirement was implicitly met by virtue of having a stand-alone system. Interviews with individual coordinators highlighted training issues and terminology, along with the curation process, as barriers to ending dual entry.

This deficiency, along with a request for additional user roles (read-only) and additional reporting capability was requested. In early February it was agreed that additional development would take place, requiring three months (Feb-Apr) of development time. The completion of the transition was pushed back to May 2007, and dual entry continued.

In April, the agreed upon modifications were released and presented at the spring semi-annual meeting. In May, 2007, all six divisions were fully using the system. They voted to end dual entry after two more weeks.

## Lessons Learned

The process of determination of requirements, migration of the legacy data, and finally fully adoption of the new system for use took a total of three years. We attributed the problems that we encountered to seven of the nine potential problem areas described by Lorenzi and Riley.

### Communications Issues

1. More face to face meetings could have saved time in the development of the data model at the start of the project.

2. A list-serve for information technology staff at each division was set up and helped to communicate IT issues during development.

### Cultural Issues

1. Lack of familiarity with centralized database technology and HTML based data entry led to difficulties in specifying system requirements. The skill set for data entry personnel for the network had to transition from reliance upon the ability to organize spreadsheet information and paper files to reliance upon the ability to navigate data entry and reporting using HTML based forms.

2. Hostility to the concept that all data would be stored at one division site delayed data migration. A third party service was investigated but was found to be too costly. Encouraging a greater sense of success of the community as a whole over the achievements of any one division might have helped to alleviate hostility up front.

3. The CHTN had been functioning long before the introduction of standards in syntactics and semantics into tissue collection. Therefore, the importance of these issues in driving the new disease terminologies was not appreciated by the primary user community (the data coordinators). In addition, the implementation of these new terms led to a loss of sense of ownership over the new system. The simplest approach was to provide a search engine to the coordinators. The coordinators could control the complexity of their searches and thus control the number of retrieved items, depending upon how broad of a search they wanted. When the user selected a retrieved item, the software would automatically generate a new tissue request and fill in the terms from the retrieved item, saving additional work for the coordinators.

Although there was not sufficient funding to create it at the time, development of an interface that is adaptable to the skill level of the user with respect to histopathological terminology could address this issue. Older terms would be treated as synonyms for the terms that they map to, and would retrieve the appropriate terminology to use in the tissue request. Similarly, information in tissue request reports could be constructed using the synonyms.

### Scope Creep

1. There was an ongoing urge to re-design the system each time a new version was available for testing. The June 2004 meeting and the working documents created as a result of the meeting were critical to containing scope creep.

2. There were unrecognized requirements resulting from migrating from stand-alone databases to centralized database. These ranged in complexity, and resulted in expanding the scope of the project.

### Leadership Issues/Organizational Issues

1. The independence of the divisions within the network required a new way of thinking for the CHTN to standardize procedures and input style.

2. Although the Informatics Subcommittee existed as part of the governing structure, a centralized Informatics Core with a mandate to implement change and support standards was lacking. This contributed to a lack of a vision for change. This was attributed to a perception of loss of ownership of the informatics to a central group.

### Lack of Sufficient Training

1. The community was engaged in testing the software in order to simultaneously train them on its use. Each division was asked to identify a "superuser" for training who could be a local resource. A demonstration site was and is still maintained for the divisions to use to train new personnel.

2. The use of dual entry to transition to the new system had a negative impact on training. Divisions delayed curating their tissue requests because they could continue working around the old system instead of learning the new system.

## Conclusion

Post-migration acceptance is high for the system overall. The most frequent complaint is that the ontology and terminology for the anatomic sites and disease lists are too complex. Constraints by the tissue user community outside of the CHTN in defining standards for disease and anatomic site classification and terminology have made it more difficult for the non-medical staff to use the system, and have also introduced a perception of loss of ownership of terminology.

Familiarity with medical terminology is becoming an increasingly important skill for tissue core managers. A very important lesson for the biomedical research community here is to understand the difficulty faced by tissue collection personnel in order for tissue collection information to be compliant with emerging standards in terminology. Tissue collection personnel must become familiar with disease terminology at the level required by organizations such as the College of American Pathologists and the National Library of Medicine. A concept based interface that guides users with different backgrounds in selecting the values for their tissue requests would address this problem.

Second to the cultural problem of adopting a new terminology, the next greatest problem encountered was the decision adopted by the community to double enter data into the old and new system prior to making a complete transition to the new system. This approach is commonly adopted in the biomedical research community with the hope that data will not be lost if the new system is not adequately debugged. As long as there is sufficient software testing across the community prior to adopting the new software, then we would recommend that double entering of data not be adopted during the course of change management. The familiarity with the old system biases the users to stay with the old system and even works to hold them back from learning how to use the new system. In this case, the difficulties encountered with migration of the legacy data, which contained unstructured entries in non-standard locations in the old database, resulted in many of the migrated entries being incorrect. Each active request had to be curated to determine its accuracy. While this resulted in a positive data clean-up step, it did introduce a lag time between migration and complete usability of the system. Given that this curation step was required, dual data entry was adopted and contributed to a delay in full adoption of the system. If dual data entry can be avoided during change management, it is our recommendation not to adopt dual data entry as a means of easing the community into adopting the new software.

Scope creep threatened to be a major problem. However, the early meeting use of a CMM level 3 equivalent approach and define requirements was a critical step that helps to minimize scope creep.

On-going review of the reasons for failure listed by Lorenzi and Riley were introduced to the Informatics Subcommittee and the user community in order to encourage the network members to work together to prevent a failure. This helped to give the community ownership of managing the change itself. In their book on managing technological change, Lorenzi and Riley spoke of how readiness for change factors into acceptance[9]. While the CHTN was ready for a change in the system that it used, and this has helped to make the transition successful, the tissue collection personnel were not affected by the issues that were motivating the adoption of terminology standards. As tissue resources at institutions across the United States migrate to more complex, standardized systems constructed for tissue and information exchange [10], it is important to consider the experiences described in this case study.

## Abbreviations

caBIG^®^: Cancer Bioinformatics Grid; CHTN: Cooperative Human Tissue Network; NCI: National Cancer Institute

## Competing interests

The authors declare that they have no competing interests.

## Authors' contributions

MEE conceived of the study design, interpretation, and preparation of the manuscript. WEG and MKW both contributed to the preparation of the manuscript for important intellectual content. All authors have read and approved the final manuscript.

## Pre-publication history

The pre-publication history for this paper can be accessed here:

http://www.biomedcentral.com/1472-6947/10/32/prepub
